# Play-by-Play Network Analysis in Football

**DOI:** 10.3389/fpsyg.2019.01738

**Published:** 2019-07-25

**Authors:** Florian Korte, Daniel Link, Johannes Groll, Martin Lames

**Affiliations:** Chair of Performance Analysis and Sports Informatics, Technical University of Munich, Munich, Germany

**Keywords:** performance analysis, football, temporal networks, flow centrality, intermediary player

## Abstract

This study identifies dominant and intermediary players in football by applying a play-by-play social network analysis (SNA) on 70 professional matches from the 1. and 2. German Bundesliga during the 2017/2018 season. SNA provides a quantification of the complex interaction patterns between players in team sports. So far, the individual contributions and roles of players in football have only been studied at match-level considering the overall passing of a team. In order to consider the real structure of football, a play-by-play network analysis is needed that reflects actual interplay. Moreover, a distinction between plays of certain characteristics is important to qualify different interaction phases. As it is often impossible to calculate well known network metrics such as betweenness on play-level, new adequate metrics are required. Therefore, flow betweenness is introduced as a new playmaker indicator on play-level and computed alongside flow centrality. The data on passing and the position of players was provided by the Deutsche Fußball Liga (DFL) and gathered through a semi-automatic multiple-camera tracking system. Central defenders are identified as dominant and intermediary players, however, mostly in unsuccessful plays. Offensive midfielders are most involved and defensive midfielders are the main intermediary players in successful plays. Forward are frequently involved in successful plays but show negligible playmaker status. Play-by-play network analysis facilitates a better understanding of the role of players in football interaction.

## Introduction

Football teams are described as groups that interact in a dynamic and interdependent way to achieve their common goal (Ribeiro et al., 2017). Understanding the individual role of each player in that dynamic process is highly relevant to uncover how a team operates (Vilar et al., 2013). Although collective behavior within teams is frequently linked to performance outcomes in sports, the impact of individual players on team performance requires further research (Duch et al., 2010). Therefore, identifying methods that offer a quantification of crucial players in the interaction of teams contributes to performance analysis in football.

Social network analysis (SNA) has been identified as a suitable method as it addresses the interdependencies in teams by modeling the interaction based on passes. Passos et al. (2011) describe the potential of SNA by modeling intra-team coordination as the frequent passing interaction taking place between players in team sports. Pena and Touchette (2012) and Grund (2012) build on this idea by connecting network properties to performance outcomes in football. Since then, there has been a growing body of research applying SNA by exploiting passing networks to understand the properties of team performance and the underlying individual contribution of players (Sarmento et al., 2018). The latter is of interest as each player has a specific position to play and role to accomplish in order to contribute to the common goal of winning (Bourbousson et al., 2010). The majority of research in football follows a static analysis on match-level by calculating centrality metrics based on the aggregated passing data in a match. In these studies, the contribution of players to the overall team performance is often described by counting the total number of successfully played and received passes through different degree measures (Clemente et al., 2015; Gama et al., 2015; Trequattrini et al., 2015). Moreover, the intermediary role of players to connect their team mates as bridging players by distributing the ball is frequently assessed by applying betweenness and closeness measures to the overall passing interaction between players across a match (Clemente et al., 2015, 2016a; Aquino et al., 2018; Castellano and Echeazarra, 2019).

Based on these existing studies that apply SNA in football, Ramos et al. (2018) demand a breakdown of the analysis to a play-by-play level to consider the temporal character of football. This implies that passing sequences should be evaluated separately instead of examining the aggregated passing data across a match. Moreover, they emphasize that an analysis on match-level to detect intermediary players through the application of betweenness and closeness measures assumes certain properties about interplay in football that might not be adequate, e.g., the proposition that ball flow follows the shortest paths over the graph which results from the aggregation of all passes in a match. That means that the current approaches do not actually consider the actual sequence of ball passing in order to detect players that are in fact connecting their team members through passing. Instead, the overall intensity of passing across a match is used to approximate bridging players. Third, the authors also suggest a distinction between plays of certain characteristics to ensure a qualitative component to the analysis that bridges the gap between SNA and performance outcomes and fosters the practical impact of the approach.

Some studies already tackle certain aspects of the proposition. Yamamoto and Yokoyama (2011) break down matches in time intervals to meet the temporal character of football. Pina et al. (2017) differentiate between successful and unsuccessful interaction based on aggregated passing networks during certain time intervals. Yet, these approaches do not reflect actual interplay as the analysis is built on aggregated passing data across a number of plays and hence does not consider actual interplay as it unfolds. The reason why most studies conduct an analysis on interval-level instead of play-level is due to the character of plays in football and the current limitations of SNA in sports. In a study by Tenga et al. (2010), 50% of all plays consist of two passes or less and only 20% of all plays take more than four passes. Thus, only a limited number of players are involved in individual plays and it is often not possible to calculate well known individual metrics such as betweenness or closeness on that level of analysis. Moreover, until recently, the regular availability of action feeds in professional football that enable a play-by-play network analysis was limited. In a recent study, Mclean et al. (2018) compute SNA metrics on play-level by analyzing the team interaction properties of goal scoring networks and modeling zones on the playing field as separate nodes to assess how attacks evolve across the pitch. However, there is no differentiation between successful and unsuccessful plays and no assessment of the contribution of individual players.

To summarize, previous research in football has not identified the individual contribution and especially the intermediary role of players based on a separate evaluation of passing sequences. Studies were only executed based on aggregated passing data across time intervals or the entire match. Thus, this study applies and proposes adequate metrics that quantify individual performance on play-level while connecting the results to performance variables. Moreover, a distinction between dominant and intermediary players on play-level is provided. Building on Clemente et al. (2016b), dominant players on match-level are frequently involved in interplay while intermediary players link other teammates during a match.

Following Fewell et al. (2012), flow centrality is calculated to assess the individual dominance on play-level by focusing on the overall involvement during all plays in a match. The intermediary role of players is quantified by counting the share of plays in which the players are actually in-between other teammates. The metric which we call flow betweenness considers the actual sequential pattern of passing and overcomes the issue of short plays, in terms of number of passes, at the same time. We draw a comparison of network metrics between different playing positions as the applied metrics specify and extend the characterization of roles and tasks of players in football. There is also a differentiation between successful and unsuccessful plays by using the entering of the finishing zone as a proxy for goals scored to achieve a rigorous assessment of individual contribution (Tenga et al., 2010). Additionally, the study draws a comparison to the traditional playmaker indicator of weighted betweenness which is computed at match-level. Using a correlation analysis, we can investigate the degree of similarity between flow-based and common match-level metrics and the circumstances in which the results between flow-based metrics differ.

The novelty about this study is twofold. First, it proposes the breakdown of a football match in its sequential order of passing within ball possessions in order to find actual bridging players that are in-between plays. Therefore, our contribution does not lie in the observation of changes in the pattern of interplay across a match but in the consideration of the temporal order of passes within plays to detect actual intermediate players. The second novelty is a comparison between the network metrics of different playing positions in successful and unsuccessful plays to assess their contribution to the team. We focus on the different outcomes of a play, instead of only assessing successful play outcomes such as Mclean et al. (2018) did or relating individual match-level metrics to match outcomes which accepts potential noise in the analysis. Flow-based metrics quantify the proportional prevalence or intermediary role of players in a match. They appear most fitting in a football context as they are robust to the short plays in football, allow a consideration of the temporal order of passing as proposed through flow betweenness and offer a suitable connection to performance outcomes on play-level.

## Materials and Methods

### Samples

A total of 70 matches between 35 professional male football teams from the 1. and 2. German Bundesliga were analyzed during the 2017/2018 season. Matches were randomly selected from a pool and, on average, teams were present in four matches with no repetition of any encounter. The final sample consists of 24,990 passes captured in 5409 plays.

### Procedure

**FIGURE 1:**
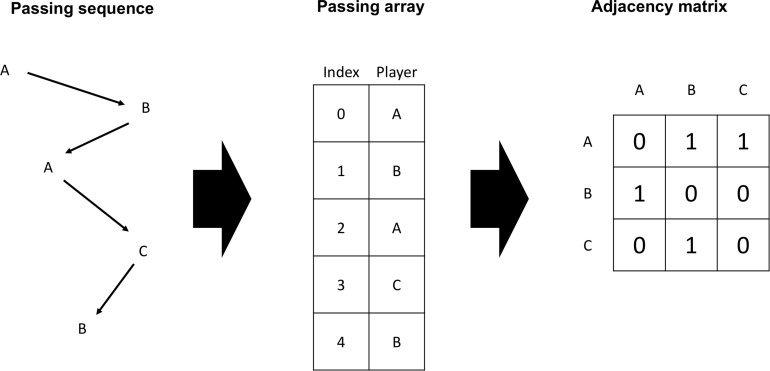
Example of a passing sequence with its corresponding passing array and adjacency matrix.

The focus of this study lies on an analysis at play-level. This means that interplay in each ball possession is examined separately instead of evaluating an aggregated passing matrix at match-level. The data was provided by the Deutsche Fußball Liga (DFL). It contains positional data for each player and the ball, which was collected by the multiple-camera tracking system TRACAB^®^ operating at 25 Hz. The validity and reliability of the system was secured in an independent study (Linke et al., 2019). Action feeds including information on passing were also provided and their reliability secured by the DFL. Definitions and validation procedures can be found in the DFL definitions catalog for official match data (2014). Twenty-eight percent of the original data is dropped in the cleaning process providing 8897 plays that clearly identified each ball possession and player involved. In order to conduct our analysis, we capture each play in a two-dimensional passing array consisting of the players in possession of the ball and an index reflecting the sequential order of the ball passing during the play. We also build a corresponding adjacency matrix for each play which are then aggregated across a match to calculate the traditional playmaker indicator on match-level. Figure 1 provides an example of a passing sequence with its corresponding passing array and adjacency matrix. For the purpose of our study, the final sample (61% of all plays) focuses on plays of at least two completed passes (the minimum play size for having an intermediate player).

We categorize a possession as successful when a team enters the finishing zone, which is a common proxy for goals scored (Tenga et al., 2010). This category includes all plays of at least two passes that lead to entering the finishing zone and sequences are captured until the moment of success (Pina et al., 2017). A play is declared as unsuccessful if ball possession is lost by any means before entering the zone. Neutral plays already start in the finishing zone or consist of set-plays directly entering it. The possession outcome was classified combining the positional data provided for each player and the ball with the information on the standardized pitch sizes in the German Bundesliga and dimensions of the finishing zone as defined by Tenga et al. (2010). The information jointly enabled an automatic evaluation on whether the player in ball possession entered the finishing zone or whether a successful pass was played to a teammate in that designated area. That way, we could also detect whether a possession starts in the finishing zone in order to declare it as neutral. This leads to 21.5% successful plays, 74.5% are declared unsuccessful and a remainder consisting of 4% in neutral plays.

Playing positions are tracked to facilitate an evaluation of the individual contribution of players in our study. Multiple players may be assigned to the same tactical position. Average metric values are reported to evaluate the performance of the playing positions in this case. The final classification is in line with previous studies focusing on players in football (Clemente and Martins, 2017; Korte and Lames, 2018). We codify the following seven playing positions according to the definitions catalog for official match data provided by the DFL (2014): (i) goalkeeper (GK); (ii) central defender (CD); (iii) external defender (ED); (iv) central defensive midfielder (CDM); (v) external midfielder (EM); (vi) central offensive midfielder (COM); and (vii) forward (F). Substitutions are handled through a reassignment of playing positions according to the DFL data provided. By codifying playing positions, in comparison to specific player tracking, there is no need to standardize the obtained values according to time on the field (Praça et al., 2019).

### Network Metrics

The analysis was carried out using the Python package NetworkX^®^ and the software libraries pandas and NumPy. A set of individual metrics was computed to achieve a quantification of the contribution of playing positions in a team’s interplay. By calculating flow centrality, a concept first introduced in basketball by Fewell et al. (2012), we capture the involvement of each playing position in all plays across a match. Building on this metric and random-walk betweenness by Newman (2005), we also compute a new metric called flow betweenness. For comparison purposes, we also calculate weighted betweenness scores for each playing position based on the aggregated passing data across a match. Whereas the two play-level metrics model pass interactions as walks, the weighted betweenness computation is based on the concept of shortest paths to evaluate the intermediary role of players (Ramos et al., 2018).

#### Flow Centrality

For each player, flow centrality measures the fraction of plays (or attack units) that it is involved in at least once relative to all plays by its team. Thus, an indication on the overall involvement of all playing positions across a match is provided. Following Fewell et al. (2012), flow centrality index, *C*_FC_(*n*_*i*_), for player *i* is calculated as,

(1) $$C_{\mathrm{FC}}\,\left(n_{i}\right)=\frac{\sum_{k=1}^{m}p_{k}\,\left(n_{i}\right)}{M}$$

where *M* denotes the total number of plays by a team in a match and *p*_*k*_(*n*_*i*_) denotes the *k*-th play in which *n*_*i*_ is part of at least once. By construction, flow centrality values are bounded between 0 and 1. The extreme value of 0 signals that a player was not part of any play in terms of passing or receiving the ball. A value of 1 means that a player was at least involved once in every play of its team during the match. Any flow centrality value in between can be interpreted as the proportion of plays that a player was involved in relative to all plays by its team.

#### Flow Betweenness

For each player, flow betweenness measures the fraction of plays in which it functions as an intermediary player relative to all plays by its team. We define a player as intermediate in a play if it actually functions as a bridging player in terms of passing between any other two players. Flow betweenness index, *C*_FB_(*n*_*i*_), for player *i* is calculated as,

(2) $$C_{\mathrm{FB}}\,\left(n_{i}\right)=\frac{\sum_{k=1}^{m}b_{k}\,\left(n_{i}\right)}{M}$$

where *M* denotes the total number of plays by a team in a match and *b*_*k*_(*n*_*i*_) denotes the *k*-th play in which *n*_*i*_ is functioning as an intermediary player. In contrast to *C*_*FC*_, which only tracks involvement, *C*_*FB*_ considers the actual passing sequence of a play to track whether a player is positioned in between a sequence to function as a bridging unit. Flow betweenness values are also bounded between 0 and 1. Values of 0 signal that a player did not once receive the ball by a teammate and successfully passed it on to another teammate in any play during a match. A value of 1 means that a player received and passed on the ball at least once in every play of its team. Values in between the extreme values are again the proportion of plays that a player functioned in as a bridging unit relative to all plays by its team.

While being *in-between* always implies being *involved* in a play, the reversal is not true. Initiating or being at the end of a play implies that a player is *involved* but not *in-between* a ball possession. Therefore, the flow centrality value of a player in a match is always at least as high as its corresponding flow betweenness value.

#### Weighted Betweenness

Weighted betweenness assesses how often a player is in-between any other two players of its team measured by their strongest passing connections across a match. Thus, its betweenness character is built on aggregated match data and does not necessarily imply that the player functioned as a bridging unit within plays. It is often used as a playmaker indicator (Pena and Touchette, 2012; Clemente and Martins, 2017). The weighted betweenness index, *C*_*WB*_(*n*_*i*_), for player *i* is calculated as,

(3) $$C_{\mathrm{WB}}\,\left(n_{i}\right)=\sum_{j\neq k\neq i}\frac{g_{j\,k}^{i}}{g_{j\,k}}$$

where $g_{j\,k}^{i}$ is the number of strongest passing connections via player *i* from players *j* to *k* and *g*_*jk*_ the total number of strongest passing connections between players *j* and *k*. The values of weighted betweenness are bounded between 0 and 1 reflecting the proportion of strongest passing connections between any two players in the network that lead via a particular player.

### Statistical Procedures

Data were analyzed for normality using Shapiro–Wilk tests. Since only 40% of data was normally distributed, non-parametric statistical analyses were used.

For both play-level metrics, multiple Kruskal–Wallis *H* test are executed to test for statistical differences between playing positions for the entire sample.

In order to differentiate between successful and unsuccessful plays, we apply Kruskal–Wallis *H* tests on two separate samples, filtering for successful and unsuccessful plays accordingly, to detect differences in play-level metrics between playing positions. Moreover, multiple Mann–Whitney *U* tests are conducted for each playing position to investigate statistical differences in metrics between the different play outcomes.

As the share of successful plays is severely higher in plays starting from the opponent’s half than from the own half of a team (28.3–16.8%), we suspect the starting half to be a moderator variable that could partly influence differences in involvement in successful against unsuccessful plays across playing positions. Hence, the same procedure to differentiate between successful and unsuccessful plays is repeated focusing on plays starting from a team’s own half. For each approach, Dunn-Bonferroni post-hoc tests offer pairwise comparisons between groups, respectively.

Our statistical analysis is conducted at a 5% significance level. Following Ferguson (2009) and Cohen (2008), non-parametric estimates of η^2^ are reported to interpret the effect size according to the following criteria: no effect (η^2^ < 0.04); small effect (0.04≤η^2^ < 0.25); moderate effect (0.25≤η^2^ < 0.64); strong effect (η^2^≥0.64). Ninety percentage confidence intervals for η^2^ are calculated following Hopkins (2017).

To assess the relationship between the network metrics, a correlation analysis is carried out across the sample. First, the Pearson correlation coefficients between *C*_*FC*_ (*C*_*FB*_) and *C*_*WB*_ are calculated, respectively to evaluate the association between metrics conducted on play-level and match-level. Second, the Pearson correlation coefficient between *C*_*FC*_ and *C*_*FB*_ is computed to assess differences between the two metrics. By construction of *C*_*FB*_, we expect the metric to be dependent on the number of passes per play. Therefore, coefficients for three subsets are calculated, following Tenga et al. (2010): (i) matches with on average less than three passes per play; (ii) matches with three to five passes per play; and (iii) matches with more than five passes per play. The strength of the correlation is assessed according to the following guide by Evans (1996): moderate (0.40≤*r* < 0.60); strong (0.60≤*r* < 0.80); very strong (0.80≤*r* < 1.0). Ninety-five percentage confidence intervals for *r* are calculated following Hopkins (2017).

## Results

### General Analysis

**FIGURE 2:**
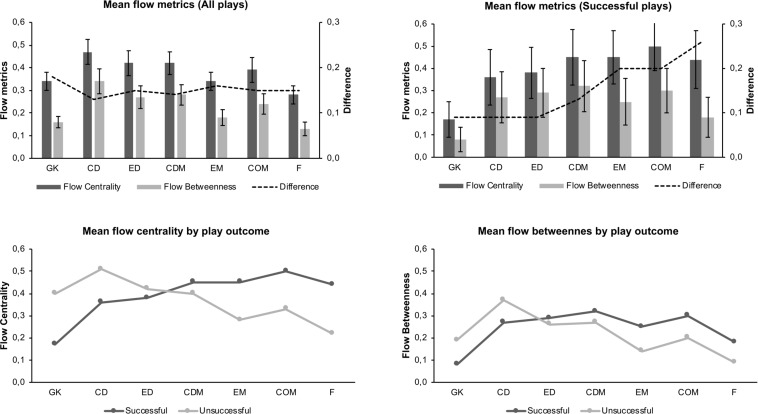
Mean results of flow-based metrics by playing position.

We find significant differences between playing positions for (*p* < 0.001;η^2^ = 0.23,*CI*[0.12,0.34],*smalleffect*) and *C*_*FB*_(*p* < 0.001;η^2^ = 0.34,*CI*[0.17,0.51], moderate effect). Figure 2 shows that CDs are significantly more involved (47% of all plays) and also function more often as intermediators (28%) in a match than any other tactical position. Fs are least involved in plays (28%) and take on an intermediary role in 13% of all attack units. By definition of the metrics, the *C*_*FB*_ value is lower for each playing position than its corresponding *C*_*FC*_ value. The largest difference between both metrics is reported for the GK.

### Success Analysis

Table 1 presents the results of the Kruskal–Wallis *H* tests for *C*_*FC*_ and *C*_*FB*_ differentiating between successful and unsuccessful plays of the overall sample and focusing on plays starting from a team’s own half. All eight tests reveal statistically significant differences between playing positions for the respective subsample with varying effect sizes.

**TABLE 1:** Kruskal–Wallis *H* test results for playing position comparison per play outcome.

	**Successful plays**				**Unsuccessful plays**			
	***H***	***p***	***η*^2^**	**CI of *η*^2^**	***H***	***p***	***η*^2^**	**CI of *η*^2^**
***C*_*FC*_**								
All plays	172.77	<0.001	0.12	[0.06, 0.18]	509.25	<0.001	0.37	[0.19, 0.55]
Own half	35.3	<0.001	0.03	[0.02, 0.04]	455.77	<0.001	0.39	[0.20, 0.39]
***C*_*FB*_**								
All plays	164.83	<0.001	0.12	[0.06, 0.18]	571.58	<0.001	0.42	[0.21, 0.63]
Own half	63.34	<0.001	0.05	[0.03, 0.07]	422.16	<0.001	0.36	[0.18, 0.54]

Table 2 presents the results of the Mann–Whitney *U* tests for each flow-based metric, playing position and differentiating also between the overall sample and focusing on plays starting from a team’s own half. Apart from the ED position, the tests reveal significant differences between successful and unsuccessful plays in terms of *C*_*FC*_ and *C*_*FB*_ for all other playing positions. However, some effect sizes are small to negligible.

**TABLE 2:** Mann–Whitney *U* test results for play outcome comparison per playing position.

	**Play outcome**						
	**GK**	**CD**	**ED**	**CDM**	**EM**	**COM**	**F**
***C*_*FC*_**							
**All plays**							
*H*	2516.5	30081	11691	31305	4070	2879	11747
*p*	<0.001	<0.001	0.090	<0.001	<0.001	<0.001	<0.001
*η*^2^	0.41	0.12	0.01	0.02	0.18	0.2	0.24
CI of *η*^2^	[0.21, 0.61]	[0.06, 0.18]	[0.00, 0.02]	[0.01, 0.03]	[0.09, 0.27]	[0.10, 0.30]	[0.12, 0.36]
**Own half**							
*H*	3522	29847.5	8710	21863.5	2844.5	3366.5	8455.5
*p*	<0.001	<0.001	0.200	0.002	<0.001	0.002	<0.001
*η*^2^	0.17	0.02	0.01	0.02	0.21	0.04	0.23
CI of *η*^2^	[0.09, 0.25]	[0.01, 0.03]	[0.00, 0.02]	[0.01, 0.03]	[0.11, 0.31]	[0.02, 0.06]	[0.12, 0.34]
***C*_*FB*_**							
**All plays**							
*H*	4129	35378.5	12186	32642.5	5275	3888.5	20974.5
*p*	<0.001	<0.001	0.230	0.005	<0.001	<0.001	<0.001
*η*^2^	0.24	0.07	0.01	0.01	0.08	0.09	0.04
CI of *η*^2^	[0.12, 0.36]	[0.04, 0.10]	[0.00, 0.02]	[0.00, 0.02]	[0.04, 0.12]	[0.05, 0.13]	[0.02, 0.06]
**Own half**							
*H*	3723.5	30416	8928	21868	4082.5	3685.5	16594.5
*p*	<0.001	<0.001	0.310	0.002	<0.001	0.025	0.017
*η*^2^	0.15	0.02	0.01	0.02	0.08	0.02	0.01
CI of *η*^2^	[0.08, 0.22]	[0.01, 0.03]	[0.00, 0.03]	[0.01, 0.03]	[0.04, 0.12]	[0.01, 0.03]	[0.00, 0.02]

In general, offensive positions (EMs, COMs, Fs) are significantly more involved in successful than in unsuccessful plays, whereas defensive positions (GK, CDs) are significantly less involved in successful plays. The *C*_*FC*_ and *C*_*FB*_ values per playing position for each play outcome and the results of the post-hoc tests can be taken from Table 3. COMs have the highest involvement in successful plays (50%) while GK take only part in 17% of all successful plays. CDs are not only most prevalent in unsuccessful plays (51%), followed by GK and EDs, but are also in-between most unsuccessful plays (37%). In contrast, CDMs are the leading intermediary players (32% of all successful plays), while GK and Fs have the lowest values in this category.

**TABLE 3:** Descriptive statistics and post-hoc results of *C*_*FC*_ and *C*_*FB*_.

	**GK**	**CD**	**ED**	**CDM**	**EM**	**COM**	**F**
***C*_*FC*_**							
**All plays**							
General	0.34 (0.08)^b,c,d,g^	0.47 (0.11)^all^	0.42 (0.11)^a,b,e,g^	0.42 (0.10)^a,b,e,g^	0.34 (0.08)^b,c,d,g^	0.39 (0.11)^b,g^	0.28 (0.08)^all^
Successful	0.17 (0.16)^all/yes^	0.36 (0.25)^a,d,e,f/yes^	0.38 (0.23)^a,f/no^	0.45 (0.25)^a,b/yes^	0.45 (0.24)^a,b/yes^	0.50 (0.22)^a,b,c/yes^	0.44 (0.26)^a/yes^
Unsuccessful	0.40 (0.12)^b,e,f,g/yes^	0.51 (0.15)^all/yes^	0.42 (0.14)^b,e,f,g/no^	0.40 (0.13)^b,e,f,g/yes^	0.28 (0.12)^a,b,c,d,g/yes^	0.33 (0.12)^a,b,c,d,g/yes^	0.22 (0.11)^all/yes^
**Own half**							
Successful	0.29 (0.27)^b,d,e,f,g/yes^	0.43 (0.32)^a/yes^	0.38 (0.31)^e/no^	0.44 (0.31)^a/yes^	0.52 (0.32)^a,c/yes^	0.42 (0.31)^a/yes^	0.47 (0.32)^a/yes^
Unsuccessful	0.48 (0.15)^d,e,f,g/yes^	0.52 (0.17)^c,d,e,f,g/yes^	0.41 (0.15)^b,e,f,g/no^	0.37 (0.15)^a,b,e,g/yes^	0.27 (0.13)^a,b,c,d,g/yes^	0.32 (0.13)^a,b,c,g,f/yes^	0.20 (0.12)^all/yes^
***C*_*FB*_**							
**All plays**							
General	0.16 (0.05)^b,c,d,f^	0.34 (0.11)^all^	0.27 (0.10)^a,b,e,g^	0.28 (0.09)^a,b,e,f,g^	0.18 (0.07)^b,c,d,f,g^	0.24 (0.09)^a,b,d,e,g^	0.13 (0.06)^b,c,d,e,f^
Successful	0.08 (0.11)^all/yes^	0.27 (0.23)^a,g/yes^	0.29 (0.22)^a,g/no^	0.32 (0.23)^a,g/yes^	0.25 (0.21)^a,g/yes^	0.30 (0.20)^a,g/yes^	0.18 (0.18)^all/yes^
Unsuccessful	0.19 (0.09)^b,c,d,e,g/yes^	0.37 (0.15)^all/yes^	0.26 (0.13)^a,b,e,f,g/no^	0.27 (0.13)^a,b,e,f,g/yes^	0.14 (0.09)^a,b,c,d,f/yes^	0.20 (0.11)^b,c,d,e,g/yes^	0.09 (0.09)^a,b,c,d,f/yes^
**Own half**							
Successful	0.13 (0.19)^b,c,d,e,f/yes^	0.32 (0.29)^a,g/yes^	0.31 (0.30)^a/no^	0.34 (0.29)^a,g/yes^	0.33 (0.30)^a,g/yes^	0.29 (0.26)^a/yes^	0.22 (0.27)^b,d,e/yes^
Unsuccessful	0.23 (0.11)^b,e,g/yes^	0.37 (0.16)^all/yes^	0.26 (0.14)^b,c,e,f,g/no^	0.26 (0.14)^b,d,e,f,g/yes^	0.13 (0.10)^a,b,c,d,f/yes^	0.19 (0.11)^b,c,d,e,g/yes^	0.10 (0.09)^a,b,c,d,f/yes^

*Significantly different to GK^*a*^, CD^*b*^, ED^*c*^, CDM^*d*^, EM^*e*^, COM^*f*^, F^*g*^, and all at *p*<0.05; yes/no indicates whether value is statistically different to other play outcome at *p*<0.05.*

Figure 2 shows that the difference between both metric scores is increasing as more offensive the playing position is on the pitch for successful plays. Moreover, while defenders and defensive midfielders are functioning as bridging players in 70–75% of all plays they are involved in, the shares for GK and Fs are only 40–50%.

Focusing on plays starting in the own half of a team, the difference of involvement and the intermediary role between successful and unsuccessful plays is reported smaller for defensive positions in comparison to the results of all plays. This indicates that the significantly large gap is moderated by the starting half of a play. In comparison to the analysis on all plays, EMs are most involved in successful plays starting from its team’s own half and come level with the intermediary player values of the other midfield positions.

### Correlation Analysis

The Pearson correlation coefficients between each flow-based metric and the weighted betweenness scores on match-level indicate a strong positive relationship for *C*_*FC*_ (*r* = 0.68;*CI*[0.64, 0.72];*p* < 0.001) and for *C*_*FB*_ (*r* = 0.67;*CI*[0.63, 0.71];*p* < 0.001). The correlation coefficient between the involvement and intermediary metric on play-level indicates a very strong positive relationship at first sight (*r* = 0.89;*CI*[0.87, 0.90];*p* < 0.001). However, the correlation strength depends on the average number of passes in plays during a match. Whereas we find a very strong positive relationship in matches with more than five passes on average per play (*r* = 0.95;*CI*[0.93, 0.96];*p* < 0.001) and also in matches with three to five passes per play (*r* = 0.86;*CI*[0.84, 0.88];*p* < 0.001), there is only a moderate positive relationship in matches with less than three passes per play (*r* = 0.56;*CI*[0.38, 0.70];*p* < 0.001).

## Discussion

The study reveals statistical significance between playing positions in successful and unsuccessful plays in football with regard to flow centrality and the newly introduced flow betweenness. Moreover, for the majority of playing positions there are significant differences between play outcomes with regard to both flow-based metrics. Effect sizes found were small to moderate with regard to playing positions and mostly small in terms of play outcomes.

Overall involvement and the frequency of being an intermediary player is lower in successful than unsuccessful plays for defensive playing positions and the other way around for offensive positions. This turns out to be partly moderated by the origin of play on the pitch, which is incident to differences in success probability. Besides, the results offer first insights into the differences between dominant and intermediary players in football measured by the two play-level metrics.

While our analysis presents CDs as the most involved and intermediary playing position, most studies traditionally ascribe midfielders the most dominant and intermediary role in football (Cotta et al., 2013; Clemente et al., 2015, 2016b). There is also literature that positions forward (Clemente et al., 2016a) and EDs (Gama et al., 2014) as intermediary players. There are multiple reasons why our results differ from past studies aside from the fact that a different sample was considered.

First, involvement (or dominance) in interplay in football is often measured by the number of successfully played and received passes in a match in form of weighted in-degree and weighted out-degree (Clemente et al., 2016a). However, there is no information on whether the passes occurred in a limited amount of longer plays, in terms of number of passes, or frequently across a match. This implies that players with high flow centrality do not necessarily play and receive most passes during a match but are most frequently part of plays across an entire match. Therefore, the match-level metrics measure the share in a team’s total passing while the play-level metric evaluates the prevalence in plays across a match.

Second, intermediary players in football, often referred to as playmakers, have formerly been determined by how often they are on average the strongest connector between the other players based on the aggregated passing data of a match (Trequattrini et al., 2015; Arriaza-Ardiles et al., 2018). However, that does not imply that the player frequently distributed the ball between other players. In an extreme scenario, a midfielder who frequently loses a ball received by defenders and frequently wins balls from the opponent and passes it to forward positions is identified as a bridging player without ever actually connecting defense and offense during a play. Flow betweenness detects how often a player is actually in-between two other players during a play and is in fact acting as an intermediary player.

Following Fewell et al. (2012) and Ramos et al. (2018), an evaluation based on the involvement in plays, however, becomes considerably more useful when making a distinction between plays with certain characteristics. While CDs appear to be the dominant and intermediary players, the majority of plays they are part of do not enter the finishing zone. In contrast, COMs are most often part of successful plays and CDM is the most intermediary position in these situations. In general, defensive playing positions show a higher involvement in unsuccessful than successful plays. Focusing solely on plays that originate in the own half of a team offsets that difference to a certain extent. Similar to previous studies (Tenga et al., 2010; Mclean et al., 2018), the share of successful plays was higher for plays starting in the opposite half and, thus, involved more offensive playing positions. The analysis on plays starting in the own half of a team partly neutralized this imbalance. This is reflected in the small to negligible effect sizes obtained when evaluating the differences in flow-based metrics between playing positions focusing on successful plays starting from the own half. Moreover, the effect sizes for differences between successful and unsuccessful plays decreases for defensive playing positions. Apart from that, the analysis provides an insight into how attacks from a team’s own half are most frequently structured. The increased metric values of the EM position in contrast to the analysis on the total sample suggest that plays were frequently build via wing positions. Therefore, the approach of subdividing the sample into different types of plays with different outcomes provides a certain quality to the analysis that goes beyond pure prevalence in plays by offering a richer insight into the structure of plays in different contexts.

The distinction between being involved and acting as an intermediary player is recognizable when focusing the analysis on successful plays. From a pure descriptive perspective, the more offensive the playing position is located on the pitch the higher its difference between the two play-level metrics. Offensive players such as forward are often involved in successful plays, however, not in order to distribute the ball but rather to take on the role of finishing attacks. While the absolute difference between the flow-based metrics for GKs might be small, the share of plays in which they function in-between others measured against all plays they are involved in is quite low. Their task is often that of an initiator of plays rather than being a bridging player. Therefore, their intermediary status is relatively low. In contrast, CDMs are similarly often involved in successful plays as forward but have a substantially higher share of incidences in which they function as a bridging player at the same time.

The correlation analysis underlines the insights of our study, especially that (i) different results on playmakers in football might be obtained when substituting match-level with play-level metrics and (ii) a distinction between play-level metrics is necessary as they emphasize different tasks among playing positions. Ramos et al. (2018) first suggested that flow centrality might be a suitable playmaker indicator that highlights intermediary players on play-level to replace the average-based analysis provided by weighted betweenness on match-level. However, the relationship between both metrics does not suggest that the same matter is measured.

Differences between values of flow centrality and flow betweenness for playing positions are also confirmed in the correlation analysis. The overestimated intermediary role of players when simply looking at involvement instead of their in-between positioning in plays is connected with the average number of passes in plays. Shorter plays offer less situations for players to be in-between plays and, thus, a sole involvement measure might exaggerate the intermediary task of a player. Hence, flow betweenness might be a more adequate playmaker indicator.

In general, the play-by-play network analysis approach allows a more contextualized performance analysis as the role of players in passing sequences of different characteristics can be evaluated separately. Barreira et al. (2015) find that team dynamics are influenced by situational variables such as match status and halves of the match. Controlling for such variables can offer a better understanding of the involvement and intermediary role of players in specific play situations.

Our study also faces some limitations that should be addressed. First, the sample only originates from two professional football leagues and, therefore, the generalizability of our results might be limited. The concern is partly offset by the findings of Mclean et al. (2017) who do not detect significant differences in passing networks between the 2016 European football championships and COPA America football championships.

Second, the determination of playing positions might contrast the less static interpretation of roles in modern football. As we break down the analysis to individual plays, the fixed assignment of positions across a match is even more challenging. We acknowledge the occupation of different areas on the pitch and fulfilling a variety of tasks as part of the role repertoire of playing positions (Korte and Lames, 2018). Hence, the spread in metric values of some playing positions might be ascribed to the mixed role interpretation of players. However, we should stress that playing positions might be interpreted differently not only across matches but also during different phases of a match depending on the specific constraints that players face. This was not considered in the present study.

Third, this study only focuses on plays with at least two completed passes to offer a calculation of flow betweenness across all plays. A study including plays with only one pass would increase the difference between flow centrality and flow betweenness simply because it offers no in-between situations for players. In fact, the correlation coefficient between both metrics greatly decreases (*r* = 0.69) when adding plays with only one pass to the analysis. However, the weakened relationship based on plays of any length also validates the introduction of a new playmaker indicator to reflect the real structure of football on a play-by-play level.

Moreover, it should be stressed that the comparison between successful and unsuccessful plays per playing position could be partly confounded by the cutoff of the passing sequences once the finishing zone is entered. Successful plays continued on average for 0.5 passes after the outcome determination. However, a separate analysis based on the entire passing sequences shows that a wider gap in play involvement between successful and unsuccessful plays for COMs and Fs is the only substantial change.

In addition, the opponent’s strength and especially defensive actions were not considered in this study, which could potentially have an impact on the involvement of certain playing positions. Focusing on the attacking side, it should be mentioned that we did not concentrate on identifying different game styles but rather aimed at emphasizing the different roles and contributions of playing positions.

## Conclusion

This is the first study that performs a play-by-play network analysis in football differentiating between plays of certain characteristics. Moreover, a novel metric is introduced to assess playmakers on play-level as an alternative or extension to flow centrality. Only a limited connection with traditional playmaker indicators on match-level can be detected. Hence, it offers new insights and a better understanding of the roles of playing positions during plays in football.

Central defenders are identified as dominant and intermediary players, however, mostly in unsuccessful plays. COMs are most involved and CDMs function mostly as intermediary players in successful attacks. Fs are frequently involved in successful plays but take on a minor intermediary role.

The practical impact of this study is twofold. First, a playmaker indicator that focuses on actual passing sequences rather than averages across a game was applied to adequately reflect interplay in football. Second, the study provides a more sophisticated understanding of the involvement and role of players in different play situations. Apart from considering play outcome, the play-by-play network analysis approach allows the inclusion of additional situational variables that are relevant to performance in football. The insights and approach of this study could be used and applied in practical performance analysis. By tracking specific players rather than playing positions, clubs can gain a better understanding of the involvement and intermediary role of their individual players in the interplay of the team.

Future studies should continuously focus on developing new SNA-metrics that reflect actual interplay and study the impact of the opponent team on the interaction of the team in ball possession. Moreover, position-specific performance indicators could complement the current play-level approach that solely focuses on whether the finishing zone was reached.

## Data Availability

The raw datasets for this manuscript are not publicly available because data sets were collected by Deutsche Fußball Liga (DFL). Requests should be directed to FK, korte@cdtm.de.

## Ethics Statement

Since each player agreed to the video recording of matches on signing their player license, an ethics approval was not required as per applicable institutional and national guidelines. Nevertheless, all procedures performed in the study were in strict accordance with the Declaration of Helsinki as well as with the ethical standards of the local ethics committee.

## Author Contributions

FK conceived the design, was responsible for the statistical procedures and interpretation of the data, wrote the manuscript, and reached the final version of the manuscript. ML supported the development of the research design and the statistical procedures, assisted in the interpretation of the data, and reviewed the manuscript. JG preprocessed the data and supported the analysis of the matches. DL supported the preprocessing of the data and provided reviews to the manuscript.

## Conflict of Interest Statement

The authors declare that the research was conducted in the absence of any commercial or financial relationships that could be construed as a potential conflict of interest.
